# Lower bile acids as an independent risk factor for renal outcomes in patients with type 2 diabetes mellitus and biopsy-proven diabetic kidney disease

**DOI:** 10.3389/fendo.2022.1026995

**Published:** 2022-10-07

**Authors:** Xiang Xiao, Junlin Zhang, Shuming Ji, Chunmei Qin, Yucheng Wu, Yutong Zou, Jia Yang, Yuancheng Zhao, Qin Yang, Fang Liu

**Affiliations:** ^1^ Division of Nephrology, West China Hospital of Sichuan University, Chengdu, China; ^2^ Department of Nephrology, The first affiliated hospital of Chengdu Medical college, Chengdu, China; ^3^ Laboratory of Diabetic Kidney Disease, Centre of Diabetes and Metabolism Research, West China Hospital of Sichuan University, Chengdu, China; ^4^ Department of Project Design and Statistics, West China Hospital, Sichuan University, Chengdu, China

**Keywords:** bile acids, diabetic kidney disease, glucolipid metabolism, end-stage renal disease, renal outcomes, risk factors

## Abstract

**Aims:**

Abnormalities of glucolipid metabolism are critical mechanisms involved in the progression of diabetic kidney disease (DKD). Bile acids have an essential role in regulating glucolipid metabolism. This study investigated the clinicopathological characteristics of DKD patients with different bile acid levels and explored the relationship between bile acids and renal outcomes of DKD patients.

**Methods:**

We retrospectively reviewed and evaluated the histopathological features and clinical features of our cohort of 184 patients with type 2 diabetes mellitus and biopsy-proven DKD. Patients were divided into the lower bile acids group (≤2.8 mmol/L) and higher bile acids group (>2.8 mmol/L) based on the cutoff value of bile acids obtained using the time-dependent receiver-operating characteristic curve. Renal outcomes were defined as end-stage renal disease (ESRD). The influence of bile acids on renal outcomes and correlations between bile acids and clinicopathological indicators were evaluated.

**Results:**

Bile acids were positively correlated with age (r = 0.152; P = 0.040) and serum albumin (r = 0.148; P = 0.045) and negatively correlated with total cholesterol (r = -0.151; P = 0.041) and glomerular class (r = -0.164; P =0.027). During follow-up, 64 of 184 patients (34.78%) experienced progression to ESRD. Lower levels of proteinuria, serum albumin, and bile acids were independently associated with an increased risk of ESRD (hazard ratio, R=5.319; 95% confidence interval, 1.208–23.425).

**Conclusions:**

Bile acids are an independent risk factor for adverse renal outcomes of DKD patients. The serum level of bile acids should be maintained at more than 2.8 mmol/L in DKD patients. Bile acid analogs or their downstream signaling pathway agonists may offer a promising strategy for treating DKD.

## Introduction

Data collected from 142 countries comprising 97.3% of the worldwide population showed that the global prevalence of diabetes among patients with end-stage renal disease (ESRD) increased from 19.0% in 2000 to 29.7% in 2015, and that the proportion of patients with ESRD attributable to diabetes increased from 22.1% to 31.3% (1). Diabetic kidney disease (DKD) is a significant microvascular complication that has become the leading cause of chronic kidney disease and ESRD, resulting in large health and economic burdens worldwide (2–4).

The management of risk factors, such as hyperglycemia, hypertension, dyslipidemia, and the use of renin-angiotensin-aldosterone system blockers, has helped to delay the progression of DKD. Recently, new therapeutic agents, including sodium-glucose transporter 2 inhibitors, endothelin antagonists, glucagon-like peptide-1 receptor agonists, and mineralocorticoid receptor antagonists, have provided additional treatment options for patients with DKD (5). Although more treatment options are available, a significant number of patients still experience progression to ESRD. Therefore, it is urgent to actively explore the pathogenesis of DKD to find more effective intervention targets.

Abnormalities of glycolipid metabolism are crucial in the development and progression of DKD. Bile acids are the main components of bile (approximately 50% of the organic bile composition) and are mainly synthesized by the liver; furthermore, they have been confirmed to regulate glycolipid metabolism (6, 7). The improvement of glycolipid metabolism has been proven to be renoprotective; therefore, bile acids may indirectly exert renoprotective effects by improving glycolipid metabolism. Additionally, many studies have shown that bile acid signaling molecules exert metabolic effects by interacting with nuclear receptors (farnesoid X receptor [FXR], pregnane X receptor, vitamin D receptor, G-protein-coupled receptors [TGR5]), and cellular signal transduction pathways (e.g., c-Jun N-terminal kinase and extracellular signal-regulated kinase) (8). This suggests that bile acids and their analogs may exert direct physiological effects by activating receptors in other organs. Some studies confirmed that bile acid derivatives or analogs can directly act on the bile acid receptors (TGR5/FXR) of the kidney to protect the kidney (9–13). Whether improving glucose and lipid metabolism or modulating energy metabolism or directly activating renal bile acid receptors, bile acids are closely related to the prognosis of DKD patients; therefore, bile acid analogs are likely to become a new treatment for DKD.

No study has confirmed whether bile acids are associated with renal outcomes of patients with DKD. Therefore, during this retrospective cohort study, we explored whether bile acid levels could predict the renal prognosis of Chinese patients with type 2 diabetes mellitus (T2DM) and biopsy-proven DKD.

## Materials and methods

### Study design and patients

**Figure 1:**
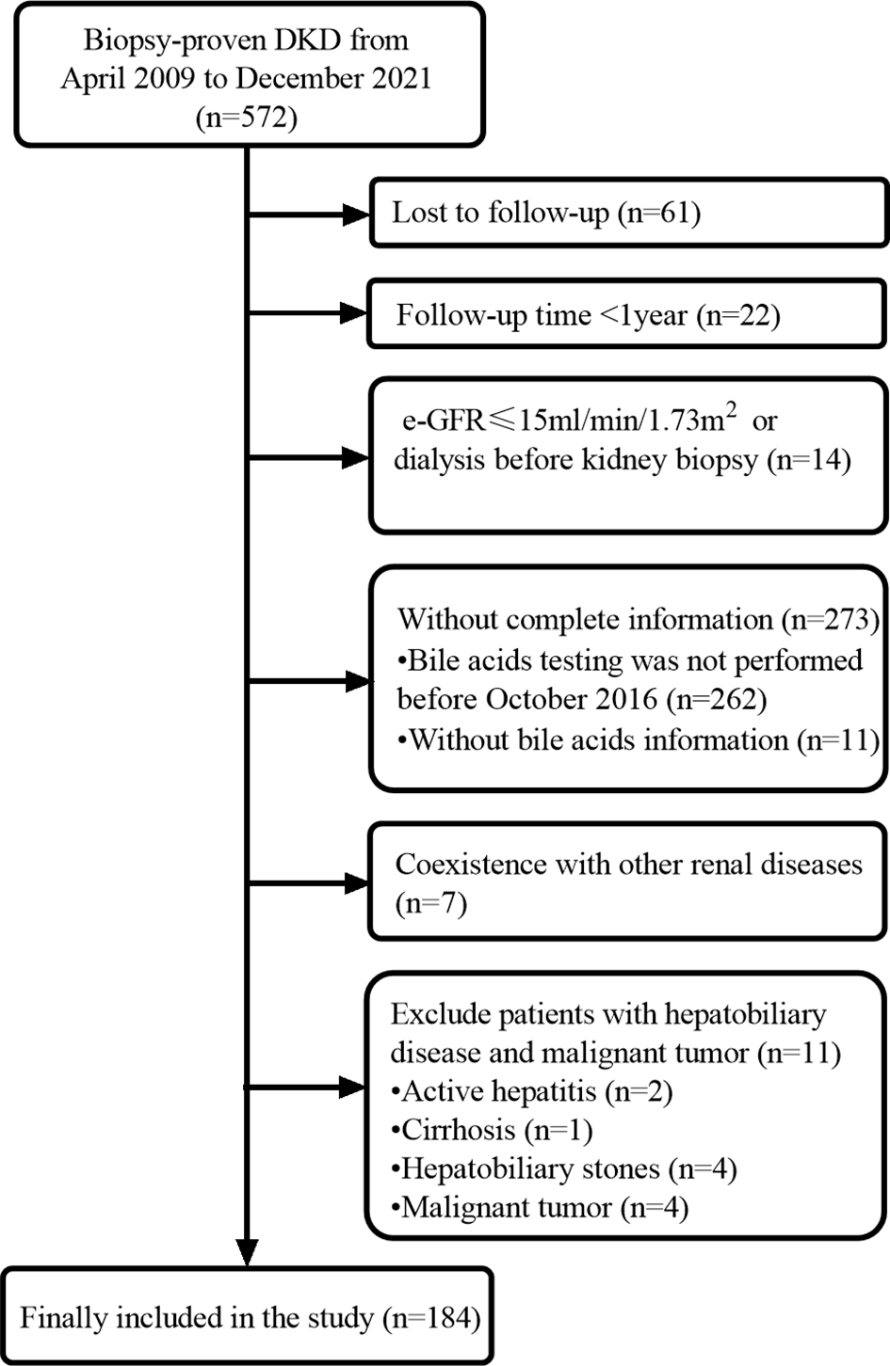
Flowchart of included patients in this study.

This was a retrospective cohort study including T2DM patients with biopsy-confirmed DKD at the West China Hospital of Sichuan University from April 2009 to December 2021. The diagnosis and classification of T2DM were based on the criteria of the American Diabetes Association (14). DKD was diagnosed according to the standards of the Renal Pathology Society in 2010. The inclusion criteria were age 18 years or older, diagnosis of T2DM, and diagnosis of DKD proven by renal biopsy. The exclusion criteria were malignant tumors, coexistence with other glomerular diseases, hepatobiliary disease (active hepatitis, cirrhosis, hepatobiliary stones), estimated glomerular filtration rate (eGFR) <15 mL/min/1.73 m^2^ or dialysis, and incomplete data (**Figure 1**). This study was approved by the ethics committee of West China Hospital of Sichuan University. The study protocol complied with the ethical standards of the 1964 Declaration of Helsinki and its later amendments. Written informed consent was obtained from all patients.

### Clinical and pathologic characteristics

Clinical and pathologic characteristics were collected from the electronic medical records at the time of renal biopsy. Subsequent follow-up evaluations of these patients were performed two to four times per year depending on the patient’s condition. The renal outcomes were defined by ESRD, which was considered the requirement for renal replacement therapy (kidney transplantation and/or hemodialysis and/or peritoneal dialysis), and/or eGFR <15 mL/min/1.73 m^2^. The eGFR was calculated using the creatinine-based Chronic Kidney Disease Epidemiology Collaboration equation. Bile acid tests were performed using an enzymatic cycling assay. All biopsy specimens were routinely examined by light immunofluorescence. The histological lesions were evaluated according to the criteria of the Renal Pathology Society (15).

### Statistical analysis

All statistical tests were analyzed using SPSS version 26.0 (SPSS Inc., Chicago, IL, USA). The normally distributed continuous variables were expressed as the mean ± standard deviation or median and interquartile range. Categorical data were presented as the number and percentage. The time-dependent receiver-operating characteristic curve (ROC) was used to evaluate the prognostic accuracy of bile acids, and the cutoff value was calculated using R4.03 (R Foundation for Statistical Computing, Vienna, Austria). When comparing two groups, we used the t test, Mann–Whitney U test, and chi-square test, as appropriate. Correlations between bile acids and clinical and pathological findings were calculated using correlation analysis. Pearson’s correlation was used for normally distributed numerical variables, and Spearman’s correlation was used for other variables. The renal survival curves were assessed using the Kaplan–Meier method and compared using the log-rank test. Cox proportional hazard models were performed to analyze the influence of bile acids on renal outcomes. A two-sided P < 0.05 was considered statistically significant.

## Results

### Baseline characteristics

**Figure 2:**
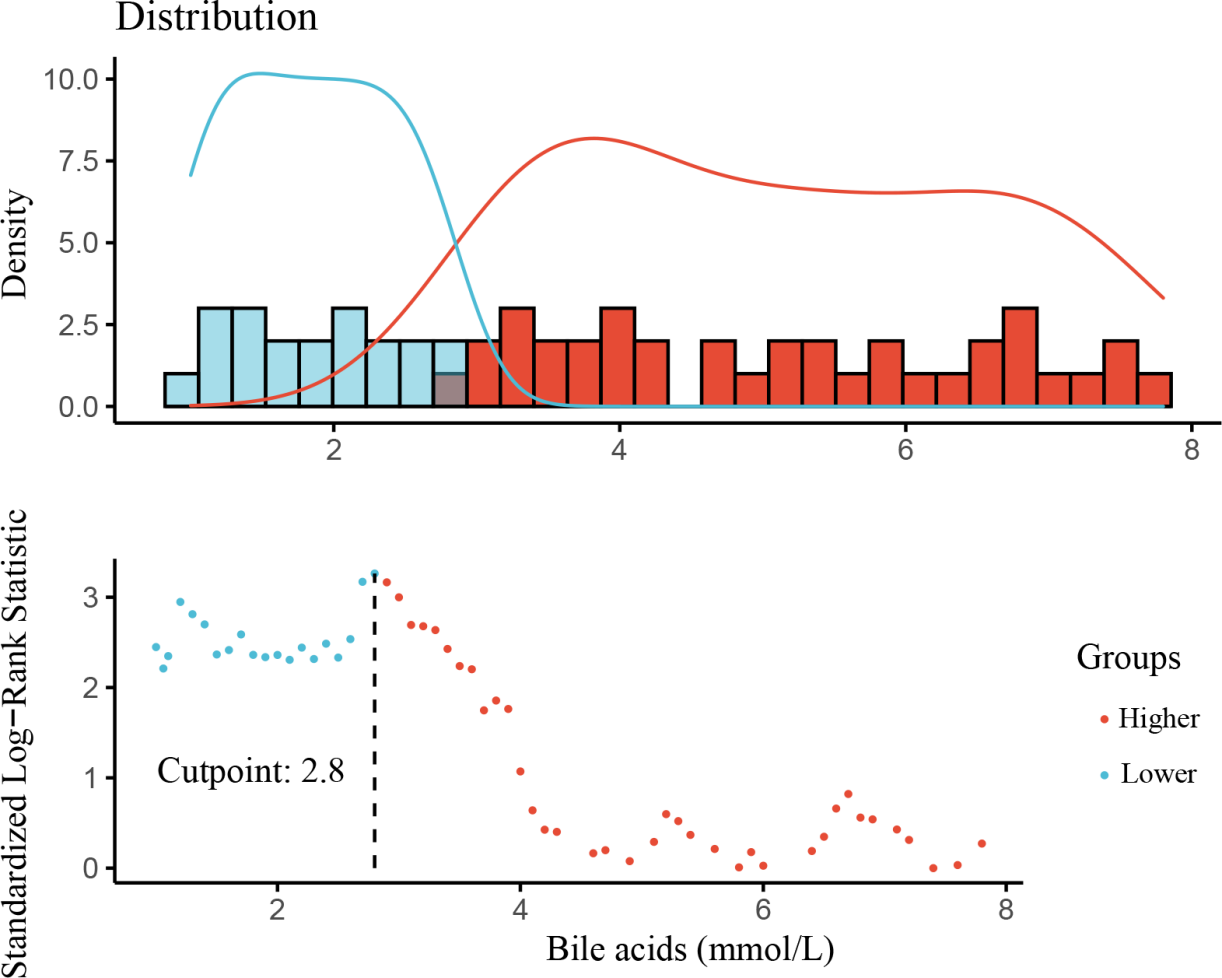
The optimal cut-point value of variables by restricted cubic spline.

This study cohort comprised a total of 184 individuals with biopsy-proven DKD (**Figure 1**). Clinical data are provided in **Table 1**. The median bile acid level was 2.80 mmol/L (1.60−4.85 mmol/L) for all patients. The median age was 51.0 years (44.0−56.0 years), and 74.5% of patients were male. The median duration of diabetes was 108.00 months (60.00−144.00 months). Complications of diabetic retinopathy were observed in 53.06% of patients. Comorbidities of hypertension were observed in 85.33% of patients. The mean proteinuria and eGFR levels were 5.16 ± 4.27 g/d and 63.21 ± 26.59 mL/min/1.73 m^2^, respectively. The patients had more severe proteinuria and lower eGFR. Furthermore, 78.0% of the patients used renin-angiotensin system inhibitors (RASI). A restricted cubic spline was used to calculate the cutoff value of bile acids (**Figure 2**). Then, patients were divided into the lower bile acids group (≤2.8 mmol/L) and the higher bile acids group (>2.8 mmol/L) according to the cutoff value. Compared with the lower bile acids group, the higher bile acids group had lower total cholesterol levels, lower low-density lipoprotein cholesterol levels, older ages, higher serum albumin levels, and higher eGFR levels (**Table 1**). There were no significant differences in the pathologic changes and use of RASI (**Table 2**).

**Table 1:** Baseline clinical features of 184 DKD patients.

Variables	All (n=184)	Lower bile acids (n=93)≤2.8mmol/L	Higher bile acids (n=91)>2.8mmol/L	p-value
Age (years)	51.00 (44.00−56.00)	50.00 (43.00−53.00)	54.5 (46.5−59.5)	0.002
Gender (male, %)	137 (74.5)	69 (74.2)	68 (74.7)	0.934
DR [n (%)]	52 (53.1)	27 (54. 0)	25 (52.1)	0.849
Duration of diabetes (Months)	108.00 (60.00−144.00)	96.00 (60.00−156.00)	108.00 (60.00−138.00)	0.936
BMI (kg/m^2^)	24.74 (22.23−26.89)	24.38 (21.51−26.53)	25.01 (22.41−27.97)	0.413
Hypertension [n (%)]	157 (85.3)	82 (88.2)	75 (82.4)	0.270
Initial proteinuria (g/day)	5.16 ± 4.27	5.45 ± 4.23	4.81 ± 4.34	0.379
e-GFR (ml/min/1.73m^2^)	63.21 ± 26.59	58.94 ± 25.30	67.46 ± 27.31	0.030
Serum creatinine (mg/dL)	133.42 ± 107.91	148.65 ± 141.96	118.02 ± 51.92	0.055
Serum albumin (g/L)	35.35 ± 7.38	33.08 ± 6.87	37.67 ± 7.20	<0.001
Hemoglobin (g/L)	120.54 ± 22.53	117.99 ± 22.51	123.15 ± 22.38	0.120
HbA1c (%)	7.89 ± 1.99	7.76 ± 2.25	8.02 ± 1.67	0.411
FBS (mmol/L)	7.15 (5.70−10.09)	7.03 (5.71−13.88)	7.71 (5.63−9.80)	0.816
Triglyceride (mmol/L)	2.12 ± 1.22	2.08 ± 1.14	2.25 ± 1.64	0.410
Total cholesterol (mmol/L)	5.08 ± 1.64	5.56 ± 1.54	4.59 ± 1.60	<0.001
LDL-c (mmol/L)	2.92 ± 1.34	3.37 ± 1.34	2.47 ± 1.18	<0.001
HDL-c (mmol/L)	1.36 ± 0.67	1.43 ± 0.73	1.28 ± 0.60	0.128
RASI [n (%)]	142 (78.0)	71 (78.0)	71 (78.0)	1.000
Progressed to ESRD (%)	64 (34.8)	40 (43.0)	24 (26.4)	0.018

DR, diabetic retinopathy; e-GFR, estimated glomerular filtration rate; FBS, fasting blood sugar; LDL, low density lipoprotein; HDL, high density lipoprotein; RASI, renin-angiotensin system inhibitor; ESRD, end-stage renal disease;

**Table 2:** Baseline pathologic features of 184 DKD patients.

Variables	All (n=184)	Lower bile acids (n=93)≤2.8mmol/L	Higher bile acids (n=91)>2.8mmol/L	p-value
Glomerular class [n (%)]				0.130
I	10 (5.4)	5 (5.4)	5 (5.5)	
IIa	38 (20.7)	14 (15.1)	24 (26.4)	
IIb	34 (18.5)	16 (17.2)	18 (19.8)	
III	74 (40.2)	39 (41.9)	35 (38.5)	
IV	28 (15.2)	19 (20.4)	9 (9.9)	
IFTA [n (%)]				0.118
0	2 (1.1)	1 (1.1)	1 (1.1)	
1	78 (42.4)	35 (37.6)	40 (47.3)	
2	74 (40.2)	36 (38.7)	38 (41.8)	
3	30 (16.3)	21 (22.6)	9 (9.9)	
Interstitial inflammation [n (%)]				0.118
0	4 (3.1)	3 (5.0)	1 (1.5)	
1	89 (70.1)	38 (60.0)	53 (79.1)	
2	32 (25.2)	20 (33.3)	12 (17.9)	
3	2 (1.6)	1 (1.7)	1 (1.5)	
Arteriolar hyalinosis [n (%)]				0.353
0	8 (5.6)	3 (4.4)	5 (6. 6)	
1	75 (52.0)	32 (47.1)	43 (56.6)	
2	61 (42.4)	33 (48.5)	28 (36.8)	

IFTA, interstitial fibrosis and tubular atrophy.

### Clinical and pathological features associated with bile acids

**Figure 3:**
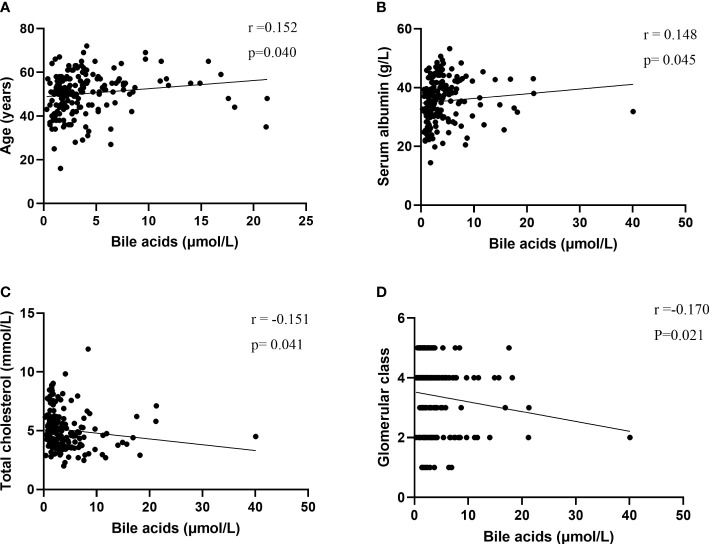
Correlations of bile acids with **(A)** Age, **(B)** serum albumin, **(C)** total cholesterol, **(D)** Glomerular class.

The bile acid level was positively correlated with age (r = 0.152; P = 0.040) and serum albumin (r = 0.148; P = 0.045) and negatively correlated with total cholesterol (r = -0.151; P = 0.041) and glomerular class (r = -0.164; P =0.027) (**Figure 3**, **Supplementary Table 1**).

### Risk of progression to ESRD

**Figure 4:**
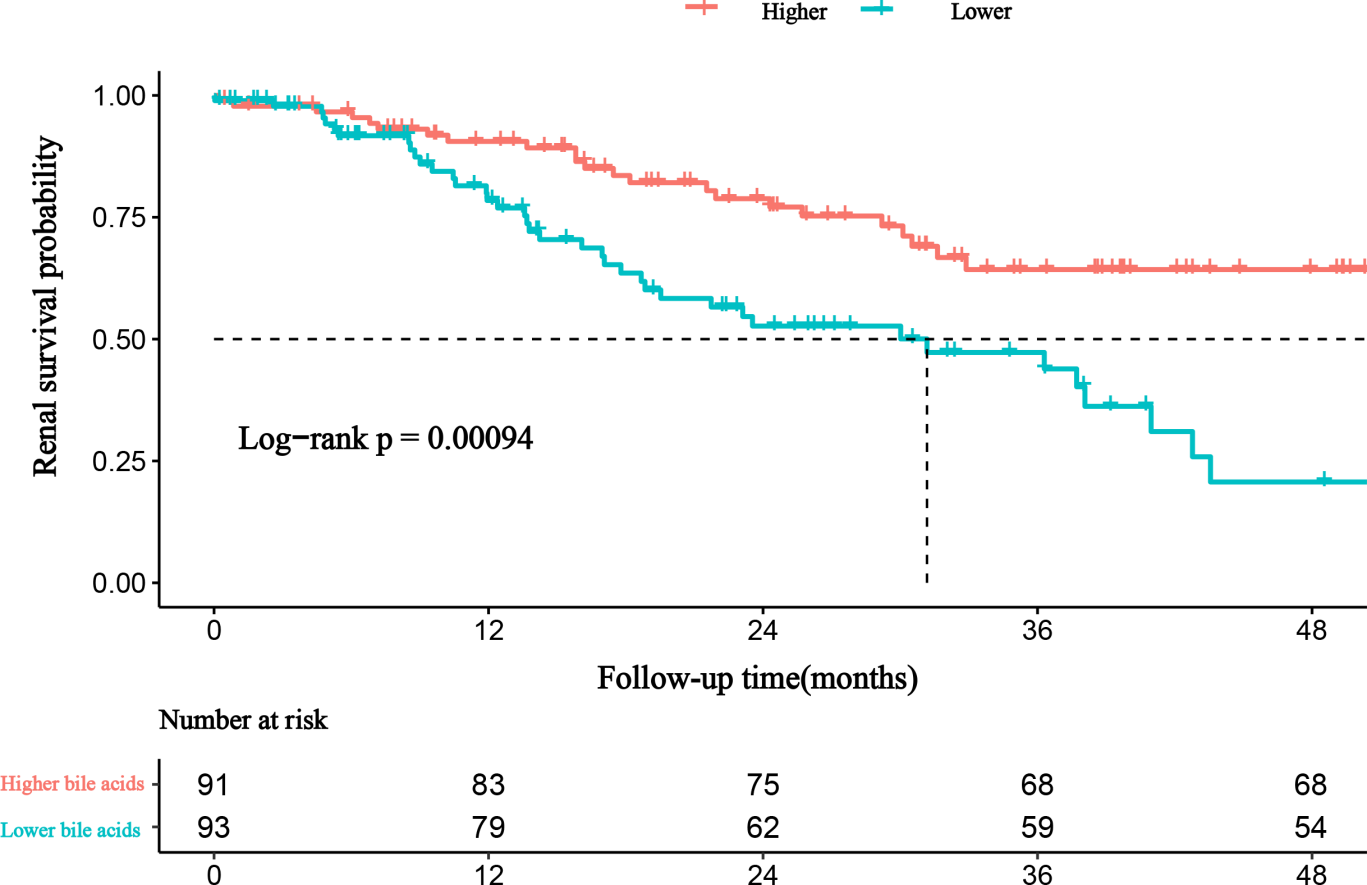
The prediction of bile acids for ESRD in DKD patients.

During the median follow-up of 19.02 months (8.65-32.39 months), 64 of 184 (34.78%) patients experienced progression to ESRD. Compared with patients with lower bile acid levels, those with higher bile acid levels were likely to have a lower incidence of ESRD (**Table 1**). A Kaplan-Meier analysis indicated that patients with lower bile acid levels at baseline were at significantly higher risk for progression to ESRD. The time-dependent ROC was used to evaluate the prognostic accuracy of bile acid levels of patients with DKD and showed that the predictive ability of bile acids for ESRD was relatively stable over time (**Figure 4**, **Supplementary Figure 1**). The Cox regression analysis evaluated the association between baseline clinicopathological variables and the renal prognosis. Univariate analyses revealed that bile acids, diabetic retinopathy (DR), body mass index (BMI), eGFR, hemoglobin, serum albumin, initial proteinuria, glomerular class, interstitial fibrosis, and tubular atrophy, and the use of RASI were risk factors for progression to ESRD (P < 0.05) (**Supplementary Table 2**). Lower bile acid levels remained independently associated with a higher risk of progression to ESRD with DKD after adjusting for baseline age, sex, BMI, DR, hypertension, DM duration, eGFR, initial proteinuria, hemoglobin, serum albumin, glomerular class, interstitial fibrosis and tubular atrophy, and RASI use (in model 3). The hazard ratio for the lower bile acids group was 5.319 (95% confidence interval, 1.208−23.425; P = 0.027) (**Table 3**). Additionally, initial proteinuria and serum albumin levels were independent risk factors for renal outcomes of patients with DKD.

**Table 3:** Associations between bile acid levels and renal outcomes.

Hazard Ratio (95% Confidence Interval)			
	Lower bile acids	Higher bile acids	p-value
	(≤2.8mmol/L)	(>2.8mmol/L)	
**Unadjusted**	2.311 (1.386-3.852)	1	0.001
**Model 1^a^**	6.006 (1.512-23.857)	1	0.011	
**Model 2^b^**	6.338 (1.555-25.834)	1	0.10	
**Model 3^c^**	5.319 (1.208-23.425)	1	0.027	

**Model 1^a^** adjusted for baseline age, gender, BMI, hypertension (yes or no), DR (yes or no), DM duration, e-GFR, and proteinuria, Hemoglobin, Serum albumin. **Model 2^b^** adjusted for covariates in model 1 plus renal pathological findings (the glomerular class, IFTA). **Model 3^c^** adjusted for covariates in model 2 plus RASI use. CI, confidence interval; DR, diabetic retinopathy; e-GFR, estimated glomerular filtration rate; IFTA, interstitial fibrosis and tubular atrophy; RASI, renin-angiotensin system inhibitor.

## Discussion

To the best of our knowledge, this is the first cohort study to relate bile acids to renal outcomes of patients with DKD. We explored the associations among bile acids, clinicopathological features, and renal outcomes of 184 patients with T2DM and biopsy-proven DKD. The results indicated that bile acids are an independent predictor of DKD progression to ESRD in T2DM patients in addition to traditional factors, including proteinuria and serum albumin levels, that serum bile acid, as a noninvasive marker, was associated with adverse renal outcomes, and that bile acid analogs and their targeting downstream signaling pathway might be promising therapeutic agents for the treatment of DKD.

Bile acids are synthesized intrahepatically from cholesterol and are the major organic component of bile. Eating foods that are high in protein can lead to increased bile acid secretion. Vagus nerve excitation can also lead to increased bile acid secretion. Humoral factors such as gastrin, pancreatin, cholecystokinin, and bile salts, can cause increased bile acid secretion. Pathological factors such as hepatobiliary disease can also lead to increased bile acid secretion. Additionally, studies have suggested that metformin (16, 17) and metabolic surgery (18, 19) increase bile acid levels.

There have been no reports of the relationship between bile acids and the renal prognosis of patients with DKD. We found that the risk of ESRD decreased with increasing bile acid levels. We obtained the cutoff value using the restricted cubic spline. Patients with DKD and bile acid levels less than 2.8 mmol/L have a poor renal prognosis. Additionally, the cutoff value is a reference value for discriminating those at clinically higher risk for ESRD. However, we believe that there is an upper limit to the bile acid level that is beneficial to renal outcomes. Exceeding the upper limit, however, may mean that more bile acid receptors will be activated and more side effects may occur, thus leading to more harm than good. The time-dependent ROC was used to evaluate the prognostic accuracy of bile acid levels of patients with DKD and showed that the predictive ability of bile acids for ESRD was relatively stable over time. The survival analysis performed during our study confirmed that patients with higher bile acid levels have a better renal prognosis. Furthermore, the risk of ESRD was 5.319-times higher for patients with lower bile acid levels compared to those with higher bile acid levels, suggesting the importance of bile acids to patient outcomes. Further exploration of the mechanisms of their protective effects is necessary.

A better renal prognosis for DKD patients with higher bile acid levels might be achieved by improvements in glucose metabolism disorders. Increasing studies have shown that bile acids are involved in glycometabolism. Wang et al. (20) demonstrated that bile acids can regulate postprandial glucose metabolism levels, suggesting a direct role of bile acids in the regulation of blood glucose. Sang et al. (21) demonstrated an increased risk of dysglycemia for Chinese community-dwelling individuals who underwent cholecystectomy, indirectly suggesting that bile acids have an important role in maintaining blood glucose. Many studies have shown that regulating blood glucose can delay DKD progression. The United Kingdom Prospective Diabetes Study was a landmark randomized, multicenter trial of glycemic therapies for 5102 patients with newly diagnosed T2DM that was conducted for 20 years (1977-1997) at 23 clinical sites in the United Kingdom and conclusively showed that intensive control can reduce the risk of microvascular complications, including progression to DKD (22–24). The ACCORD (25), ADVANCE (26), and VADT (27) studies also confirmed the same conclusion for patients with T2DM. Furthermore, a meta-study evaluated seven trials involving 28,065 adults who were monitored for 2 to 15 years and showed that compared with conventional control, intensive glucose control reduced the risks of microalbuminuria and macroalbuminuria (28). However, our study showed no correlation between bile acid levels and HbA1c and fasting blood sugar levels of patients with DKD. This may have occurred because most patients had been treated with glucose-lowering therapy. In our study, glucose-lowering therapy, including the use of insulin and oral hypoglycemic agents, was used for 89.9% of the patients. Therefore, we think that the renal protective effects of bile acids may be attributed to improved glucose metabolism.

The better renal prognosis for DKD patients with higher bile acid levels might be achieved by their improved glycolipid metabolism disorders. We initially recognized that the primary role of bile acids is to promote the digestion and absorption of lipid nutrients, thus serving as amphipathic biological detergents for lipid metabolism (6). Bile acids are the end products of cholesterol catabolism and have an important role in maintaining cholesterol homeostasis and preventing the build-up of toxic metabolites and the accumulation of cholesterol (7). However, hyperlipemia is a traditionally recognized risk factor for cardiovascular disease for patients with T2DM and DKD (29). Several studies have shown that high triglyceride levels and/or low high-density lipoprotein cholesterol levels are independent risk factors for DKD in patients with the recommended target values of blood glucose and blood pressure for type 1 diabetes mellitus and T2DM (30). Muntner et al. (31) investigated the relationship between plasma lipids and kidney disease indicated by an increase of ≥0.4 mg/dL in the serum creatinine level of the large cohort of the Atherosclerosis Risk in Communities study that included patients with T2DM. The United Kingdom Prospective Diabetes Study investigating baseline clinical risk factors associated with the later development of kidney dysfunction in more than 4000 participants, all with T2DM, identified that higher triglyceride and low-density lipoprotein cholesterol levels significantly and independently predicted incident renal impairment (32). The Early Treatment Diabetic Retinopathy Study revealed that increased serum triglyceride and total cholesterol levels were independently associated with kidney outcomes (33). The role of bile acids in regulating lipid metabolism was also confirmed during our study. We found that there was a negative correlation between bile acids and total cholesterol with DKD. Therefore, we consider that the renoprotective effect of bile acids may be attributable to the improved lipid metabolism of patients with DKD.

Bile acids may improve renal outcomes of patients with DKD by directly activating renal FXR or TGR5. In human and animal models, tubular cells and glomerular cells of the kidney highly express FXR, and FXR is downregulated in diabetic kidney disease (9). Wang et al. (10) demonstrated accelerated renal injury in diabetic FXR knockout mice. In contrast, treatment with the FXR agonist INT-747 improved renal injury by decreasing proteinuria, glomerulosclerosis, and tubulointerstitial fibrosis and modulating renal lipid metabolism. Similarly, Jiang et al. (11) reported that FXR modulates renal lipid metabolism, fibrosis, and DKD. Many studies have suggested that FXR activation inhibits inflammation in DKD (12). Moreover, FXR activation improves diabetic tubular function and tubular toxicity (34–36). TGR5 was identified as a membrane receptor for bile acids which is highly expressed in tubules, podocytes, and mesangial cells in the kidney (37, 38). It has been confirmed that the TGR5 agonist INT-777 induced mitochondrial biogenesis, decreased oxidative stress, increased fatty acid beta oxidation, and decreased renal lipid accumulation (39). We found that the bile acid level was negatively correlated with the severity of the glomerular injury, suggesting that bile acids may activate receptors and downstream signaling pathways in glomerular cells. Therefore, the direct relationship between bile acids and kidney injury must be explored.

Metformin can increase bile acid levels and the glucose-lowering effect, which may benefit the kidneys. Possible mechanisms for metformin-induced suppression of active bile acid reabsorption in the ileum are inhibition of the apical sodium-dependent bile acid transporter and modulation of the transcriptional activity of FXR via an AMPK-mediated mechanism in enterocytes (16). However, metformin is contraindicated for many individuals with impaired kidney function because of concerns of lactic acidosis (40). Nevertheless, many studies have suggested that metformin may have renoprotective effects on DKD. A recent retrospective study confirmed that metformin for advanced chronic kidney disease patients decreased the risk of all-cause mortality and incident ESRD. Additionally, metformin did not increase the risk of lactic acidosis. However, because of the remaining bias even after propensity score matching, further randomized, controlled experiments with large samples are necessary to change real-world practice (41). Therefore, metformin may exert renoprotective effects through bile acids in DKD, but the specific mechanism requires further investigation. Unfortunately, our data lacked information regarding metformin treatment, and it was impossible to analyze the relationship between metformin and bile acids during our study.

Bariatric and metabolic surgeries, including Roux-en-Y gastric bypass and vertical sleeve gastrectomy, are known to increase bile acid secretion and alter bile acid composition, particularly after Roux-en-Y gastric bypass (18, 19). The mechanisms underlying the benefits of bariatric and metabolic surgeries likely involve the bile acids signaling pathway mediated mainly by nuclear FXR and the membrane TGR5, the interaction of bile acids and gut microbiota, and exosomes (18, 19). Bariatric and metabolic surgeries have been shown to improve hyperglycemia, insulin sensitivity, and hyperlipidemia (19). These renoprotective effects may be closely related to the bile acid and glycolipid metabolic benefits associated with bariatric and metabolic surgeries. However, the effects on important endpoints of kidneys, such as ESRD and eGFR changes, must be further confirmed by randomized controlled experiments with large samples. Furthermore, the mechanism of action in DKD requires more research for further elucidation.

Higher levels of bile acids with better renal outcomes may be attributed to the indirect effects of bile acids that result in improved glycolipid metabolism and the direct effects of activating bile acids receptors to protect the kidney.

We also found a negative correlation between bile acid levels and age; this may have occurred because the synthesis and secretion of bile acids are different in individuals of different ages. We found a positive correlation between bile acid and serum albumin levels; however, more studies exploring the possible mechanism are necessary.

This study had some limitations. First, this was a retrospective study; therefore, some selection bias was inevitable. Second, the patients had biopsy-proven DKD, and the sample size was insufficient. Finally, we did not control all therapeutic interventions (such as glucagon-like peptide-1 and sodium-glucose transporter 2), which could have been confounders of the results.

In conclusion, our study describes a novel marker for predicting the renal outcomes of DKD and indicates that the serum level of bile acids should be maintained at more than 2.8 mmol/L in patients with DKD. Our study also predicted that bile acid analogs and their targeting downstream signaling pathway might be promising therapeutic agents for the treatment of DKD.

## Data availability statement

The raw data supporting the conclusions of this article will be made available by the authors, without undue reservation.

## Ethics statement

The studies involving human participants were reviewed and approved by the ethics committee of West China Hospital of Sichuan University. The patients/participants provided their written informed consent to participate in this study. Written informed consent was obtained from the individual(s) for the publication of any potentially identifiable images or data included in this article.

## Author contributions

Conception and design of the study: XX, JZ, SJ, and FL. Acquisition and analysis of data: XX, SJ, JZ, YTZ, JY, YW, YCZ, and QY. Drafting the manuscript or figures: XX, JZ, SJ, and FL. All authors contributed to the article and approved the submitted version.

## Funding

This study was supported by the Health Commission of Sichuan Province Program (no. 21ZD001).

## Conflict of interest

The authors declare that the research was conducted in the absence of any commercial or financial relationships that could be construed as a potential conflict of interest.

## Publisher’s note

All claims expressed in this article are solely those of the authors and do not necessarily represent those of their affiliated organizations, or those of the publisher, the editors and the reviewers. Any product that may be evaluated in this article, or claim that may be made by its manufacturer, is not guaranteed or endorsed by the publisher.
