# Supplementary material for: Lower bile acids as an independent risk factor for renal outcomes in patients with type 2 diabetes mellitus and biopsy-proven diabetic kidney disease
Source: Front Endocrinol (Lausanne). 2022 Oct 7;13:1026995. doi: 10.3389/fendo.2022.1026995 (PMC9585231; doi:10.3389/fendo.2022.1026995)
Supplement: Supplementary file 1 [file Table_1.docx]

**Supplementary table 1. Correlations of bile acid levels with different clinicopathological data**

| Variables | Correlation coefficient | p-value |
| --- | --- | --- |
| Age | 0.152 | 0.040 |
| Duration of diabetes | 0.059 | 0.428 |
| BMI | 0.066 | 0.378 |
| Initial proteinuria | 0.001 | 0.988 |
| e-GFR | 0.104 | 0.164 |
| Serum creatinine | -0.087 | 0.240 |
| Serum albumin | 0.148 | 0.045 |
| Hemoglobin | 0.027 | 0.713 |
| HbA1c | 0.095 | 0.237 |
| FBS | -0.065 | 0.384 |
| Triglyceride | -0.002 | 0.974 |
| Total cholesterol | -0.151 | 0.041 |
| LDL-c | -0.129 | 0.081 |
| HDL-c | -0.084 | 0.255 |
| Glomerular class | -0.164 | 0.027 |
| IFTA | -0.141 | 0.057 |
| Interstitial inflammation | -0.125 | 0.161 |
| Arteriolar hyalinosis | -0.141 | 0.091 |

e-GFR, estimated glomerular filtration rate; ESRD, end-stage renal disease; FBS, fasting blood sugar; LDL, low density lipoprotein; HDL, high density lipoprotein; IFTA, interstitial fibrosis and tubular atrophy.

**Supplementary table 2. Cox regression analysis of risk factors for renal outcomes of DKD patients**

| **Variables** | **Unadjusted** | | **Model 3** | |
| --- | --- | --- | --- | --- |
|  | **HR (95%CI)** | **p value** | **HR (95%CI)** | p-**value** |
| Bile acids | 2.311 (1.386-3.852) | 0.001 | 5.319 (1.208-23.425) | 0.027 |
| Age (years) | 0.994 (0.971-1.018) | 0.622 | 1.022 (0.948-1.103) | 0.566 |
| Gender (male, %) | 0.917 (0.700-1.202) | 0.530 | 1.553 (0.840-2.871) | 0.160 |
| DR [n (%)] | 2.938 (1.482-5.826) | 0.002 | 3.360 (0.999-11.303) | 0.050 |
| Duration of diabetes（Months） | 1.001 (0.997-1.004) | 0.690 | 1.001 (0.990-1.012) | 0.908 |
| BMI (kg/m^2^) | 0.935 (0.875-0.999) | 0.046 | 0.833 (0.664-1.044) | 0.113 |
| Hypertension [n (%)] | 0.767 (0.503-1.169) | 0.218 | 0.427 (0.111-1.640) | 0.215 |
| Initial proteinuria (g/day) | 1.148 (1.089-1.210) | <0.001 | 1.271 (1.011-1.597) | 0.040 |
| e-GFR (ml/min/1.73m^2^) | 0.970 (0.958-0.982) | <0.001 | 1.004 (0.971-1.038) | 0.812 |
| Serum albumin (g/L) | 0.886 (0.854-0.919) | <0.001 | 0.859 (0.768-0.959) | 0.007 |
| Hemoglobin (g/L) | 0.969 (0.957-0.981) | <0.001 | 1.030 (0.990-1.072) | 0.142 |
| HbA1c (%) | 0.882 (0.754-1.032) | 0.117 | 0.741 (0.530-1.036) | 0.080 |
| RASI [n (%)] | 0.501 (0.288-0.872) | 0.015 | 0.632 (0.149-2.677) | 0.534 |
| Glomerular class [n (%)] | 1.536 (1.197-1.971) | 0.001 | 1.107 (0.588-2.083) | 0.754 |
| IFTA | 1.989 (1.411-2.803) | <0.001 | 0.549 (0.213-1.419) | 0.216 |

e-GFR, estimated glomerular filtration rate; ESRD, end-stage renal disease; LDL, low density lipoprotein; HDL, high density lipoprotein; RASI, renin-angiotensin system inhibitor; RASI, renin-angiotensin system inhibitor; IFTA, interstitial fibrosis and tubular atrophy.
