# Supplementary figures and images for: Lower bile acids as an independent risk factor for renal outcomes in patients with type 2 diabetes mellitus and biopsy-proven diabetic kidney disease
Source: Front Endocrinol (Lausanne). 2022 Oct 7;13:1026995. doi: 10.3389/fendo.2022.1026995 (PMC9585231; doi:10.3389/fendo.2022.1026995)

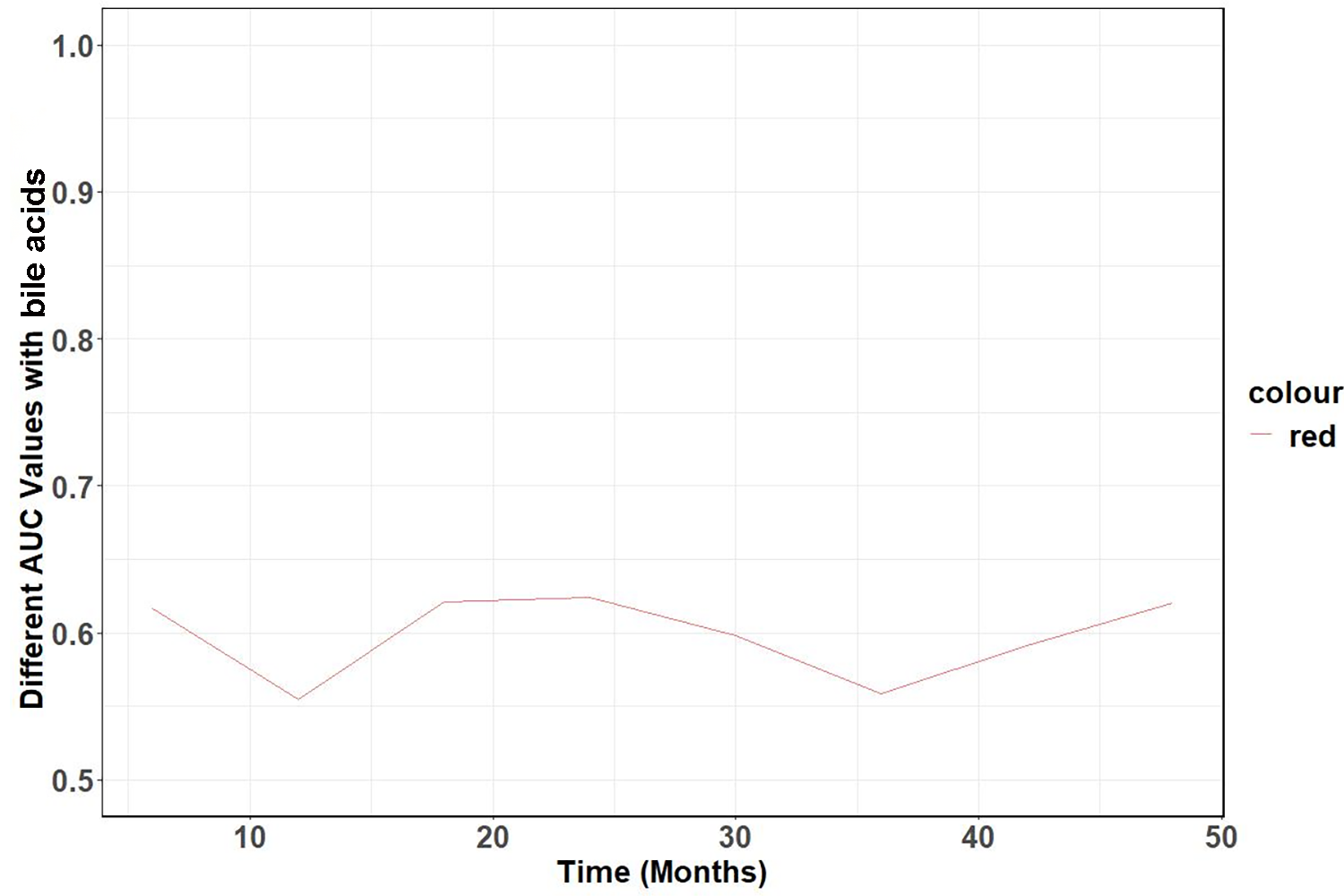

Supplement: Supplementary file 2 [file Image_1.tif]
